# The power of parenting: mitigating conduct problems among adolescents carrying genetic risk

**DOI:** 10.3389/frcha.2025.1597229

**Published:** 2025-11-06

**Authors:** Maia Choi, Genevieve F. Dash, Sally I. Kuo, Fazil Aliev, Holly E. Poore, Sarah J. Brislin, Danielle M. Dick

**Affiliations:** 1 Department of Psychology, School of Arts and Sciences, Rutgers University, NJ, United States; 2 Rutgers Addiction Research Center, Rutgers University, Piscataway, NJ, United States; 3 Department of Psychiatry, Robert Wood Johnson Medical School, Rutgers University, Piscataway, NJ, United States

**Keywords:** parenting, externalizing, polygenic risk score (PRS), conduct problem, Avon Longitudinal Study of Parents and Children (ALSPAC)

## Abstract

Conduct problems (CPs), including aggression, antisocial behavior, and rule-breaking, emerge in childhood and adolescence. Evidence from twin studies shows that CPs are heritable, with approximately 50% of the variance accounted for by genetic influences. Parenting is one prominent and, importantly, modifiable environmental factor in the development of CPs. This study tested whether parental monitoring moderated the associations between genetic liability and CPs in adolescents aged 12–14. We found parental monitoring significantly moderated the association between genetic risk for externalizing and CPs in adolescence. These findings underscore the utility of family-based prevention and intervention efforts, particularly for children at elevated genetic risk.

## Introduction

Conduct problems (CPs), including aggression, antisocial behavior, and rule-breaking, emerge in childhood and adolescence. CPs are the leading cause of mental health service referrals among youth and are associated with high social and economic burdens at the individual, familial, and societal levels in both the short and long term (1). These behaviors are also linked to a myriad of mental health sequelae, justice system involvement, and significant disruption in life opportunities, such as educational and occupational achievement, resulting in lasting downstream effects into adulthood (2). Approximately 1%–4% of children experience CPs, although retrospective studies of lifetime prevalence suggest that up to 10% of individuals display clinically significant CPs during their childhood and/or adolescence (3, 4). Despite the relatively high prevalence in childhood and adolescence, research has found that only 53% of children diagnosed with behavioral problems receive treatment (compared to 80% for children with depression) (5). Therefore, research is needed to further understand how the development of these behavioral problems can be disrupted, identifying modifiable targets for treatment and intervention. Evidence from twin studies shows that CPs are heritable, with approximately 50% of the variance accounted for by genetic influences (3, 4). However, genetic liability is not determinative; environmental influences also significantly shape outcomes by strengthening or weakening the associations between genetic liability and phenotype.

Parenting is one prominent and, importantly, modifiable environmental factor in the development of CPs. This is especially relevant during adolescence, which is a developmentally sensitive period for the effects of psychological interventions, with the potential to change the course of a developmental trajectory in high-risk individuals (6). Twin studies have demonstrated that during adolescence, parental monitoring can moderate the heritability of CPs, wherein genetic effects, as inferred via twin correlations, are reduced at higher levels of parental monitoring (7). Advances in genetics now make it possible to identify specific genetic variants from well-powered genome-wide association studies (GWASs); associated variants can be summed and weighted by their effect sizes to create a polygenic score (PGS) representing an individual's genetic liability (8). A recent multivariate GWAS used data from ∼1.5 million individuals to identify genes associated with externalizing, a constellation of disorders and behaviors characterized by behavioral disinhibition (9). The GWAS on externalizing yielded a polygenic score (EXT PGS) that accounted for nearly 10% of the variance in phenotypic externalizing (10). Furthermore, the EXT PGS robustly predicts both externalizing behaviors, such as impulsivity and drug use, and disorders, such as conduct disorder, oppositional defiant disorder, attention deficit hyperactivity disorder (ADHD), and substance use disorders (10–12). This has been found across developmental stages (e.g., toddlerhood through adulthood) in a wide range of large population-based studies and high-risk samples, and after controlling for family relatedness (10–12).

Given findings from the twin studies highlighting the importance of parenting, this study tested whether parental monitoring moderated the associations between genetic liability, as measured by PGS, and CPs in adolescents aged 12–14.

## Methods

### Sample description

The Avon Longitudinal Study of Parents and Children (ALSPAC) is a population-based, longitudinal cohort study that began in 1990 in the UK (13, 14). Pregnant women residing in Avon, UK, with expected dates of delivery between 1 April 1991 and 31 December 1992 were invited to take part in the study. The initial number of pregnancies enrolled was 14,541 and 13,988 children were alive at 1 year of age. Additional mother and child pairs that were initially eligible for the study but did not participate were enrolled when the children were approximately 7 years of age; thus, the total sample size for the analyses using any data collected after the age of 7 years is 15,447 pregnancies. Longitudinal data were collected on both the mother and offspring, starting when the mother was pregnant. Biological, psychological, health, and environmental measures, which included both parent and child reports, and genetic data are available. Since 2014, study data have been collected and managed using Research Electronic Data Capture (REDCap) tools hosted at the University of Bristol (15). The study’s website contains the details of all the data that are available in a fully searchable data dictionary and variable search tool (http://www.bristol.ac.uk/alspac/researchers/our-data/). Ethical approval for the study was obtained from the ALSPAC Ethics and Law Committee and local research ethics committees. Consent for the use of biological samples was collected in accordance with the Human Tissue Act (2004). The analyses in this project focused on the offspring from ages 6 months to 28 years who had genetic similarity to the European reference panel and available genotypic data (*N* = 8,013).

### Genetic data

DNA samples were obtained from several sources at various timepoints. Blood samples were taken from the children at ages 3, 5, and 7 years. At age 7, the participants who were unwilling to provide blood samples provided mouthwash samples for DNA extraction. Further, DNA extraction from cord blood was used for individuals who did not provide a blood or mouthwash sample. DNA samples were collected from approximately 11,000 children. The children were genotyped using the Illumina HumanHap 550 (CA, USA) quad chip genotyping platform.

### Measures

#### Conduct problems

The Development and Well-Being Assessment is a structured clinical interview designed to generate diagnostic and statistical manual of mental disorders - IV (DSM-IV) diagnoses for individuals between the ages of 5–16 years old (16). In instances of measurement during early adolescence, only 7 out of the 15 total DSM-IV symptoms of conduct disorder were assessed. CPs were measured in assessments at the ages of 10.5, 13.5, and 15.5 years. Due to the nature of the assessment schedule, the respondent’s age did not always correspond to the assessment timepoint; for example, some respondents were 12 years old at the 10.5 timepoint or 14 years old at the 15.5 timepoint. To maximize the available data, we used observations from these three timepoints but restricted the respondents to those aged 12–14 to capture data on CPs during early adolescence. Items on CPs were coded as 0 (*no*) or 1 (*yes*) and summed to generate a total CP score, with higher scores representing higher levels of CPs.

The items on conduct problems were as follows:

1. The child told lies to get things or favors from others or to get out of things they were supposed to do.
2. The child often started fights with those other than brothers and sisters.
3. The child bullied/threatened people.
4. The child stayed out much later than supposed to.
5. The child stole things from their house, other people's houses, shops, or school.
6. The child ran away from home or ever stayed away all night without the respondent's permission.
7. The child often played truant (bunked off) from school.

### Parental monitoring

The Parenting Practices Scale (17) measures youth-reported levels of parental monitoring, capturing parental knowledge, solicitation, and control and adolescent disclosure. The scale was administered to those aged between 12.5 and 13.5 years. There are a total of 24 items (e.g., “Must you have your parents’ permission before you go out during the weeknights?” and ‘‘Do your parents know what you do during your free time?’’) measured on a 4-point scale with response options ranging from “never” to “always.” The item response scores were summed to generate a scale total, with z-transformed higher scores representing higher levels of parental monitoring and knowledge.

### Data preparation

Data were prepared using R version 4.4.0. For repeated measures in the period between the ages of 12–14 years, the highest score for each variable was used in the analyses. Prorating was used for continuous measures to account for missing items. Individuals missing 50% or more of the items were coded as missing. The 52 respondents in the analytic sample who were missing more than 0% but less than 50% of the items on the CP outcome variable (∼1% of the total analytic sample) were ultimately excluded from the analysis due to prorated scoring resulting in non-integer values, which are incompatible with the analytic approaches most appropriate for modeling count variables such as the CP outcome in the present study.

### Calculating polygenic risk scores

Imputation to the 1000 Genomes reference panel and standard quality control of the single-nucleotide polymorphisms (SNPs) were performed by the ALSPAC study team. The EXT PGS was constructed from the aforementioned GWAS (10). A unified analytic pipeline was used to construct the EXT polygenic score in European-like individuals from the results of the multivariate GWAS on externalizing (10). The pipeline relied on two software packages, PRS-CS (8), to adjust the original GWAS beta weights for linkage disequilibrium (LD), and Plink2 (18), to construct the EXT PGS from the LD-adjusted beta weights. Prior to PGS construction, LD adjustment of the original GWAS beta weights was performed, as modeling LD between SNPs is known to increase the signal-to-noise ratio in polygenic scores. The 1000 Genomes European reference files distributed with the software were used as the reference panel to estimate the LD. Furthermore, as the PRS-CS method is currently restricted to the ∼1.3 million SNPs in the high-quality consensus genotype set defined by the HapMap 3 Consortium (19), polygenic scores were only generated using HapMap 3 SNPs. All other parameters were set to the default parameters in the PRC-CS software. Plink2 was used to compute the polygenic scores solely of individuals with recent European ancestry, as estimated by their genetic data, using the LD-adjusted beta weights. EXT PGS was standardized via z-transformation for analysis.

### Analyses

The analyses were conducted using SAS version 9.4. To generate descriptive statistics, “risk groups” were generated by classifying the sample into groups based on the combined level of EXT PGS and parental monitoring. PROC RANK was used to create quantiles for each variable, which were subsequently used in combination to create the following groups based on EXT PGS (low vs. high genetic risk) and parental monitoring (low vs. high parental monitoring): high–high, high–low, low–high, and low–low. Descriptive statistics for each of these groups were generated, and means were compared among the groups using t-tests. Effect sizes, as indexed by Cohen's *d*, were also calculated for each group difference.

A generalized linear model was fit using PROC GENMOD and the full analytic sample. A Poisson distribution was used to model CPs due to skewness, but not overdispersion, of the count variable. The summed CP score was regressed on EXT PGS, parental monitoring, and an EXT PGS × parental monitoring interaction term. Covariates (top 10 ancestry principal components, age, and sex) were residualized on the EXT PGS before including it in the interaction.

## Results

**Figure 1:**
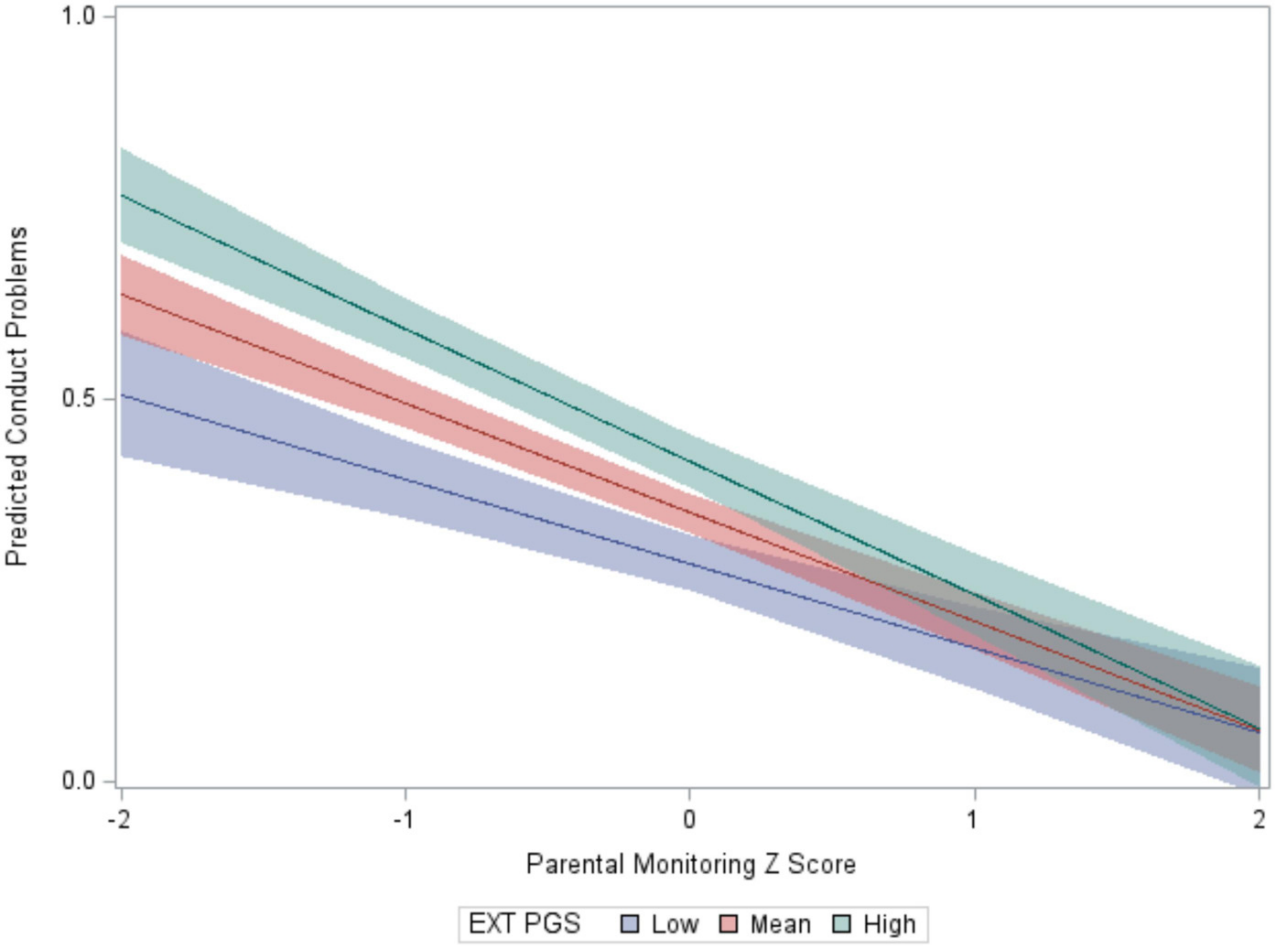
Interaction between the EXT PGS and parental monitoring in the prediction of conduct problems. Note. To aid visualization, the EXT PGS was categorized as low (below 1 SD), mean, and high (above 1 SD); the *y*-axis is on a log count scale.

The CP score mean of the analytic sample (*N* = 4,303) was 1.45 (SD = 0.89; range = 0–7). The “highest risk” group (high EXT PGS, low parental monitoring) reported more CPs than the other groups (Table 1). A large effect was observed for parental monitoring (high vs. low) in the presence of high EXT PGS (*d* = 0.70), while a small effect was observed for EXT PGS (high vs. low) in the presence of high parental monitoring (*d* = 0.11). This pattern was supported by the regression model. In addition to the significant main effects of EXT PGS and parenting (Table 2), there was a significant interaction between EXT PGS and parenting in predicting CPs, with fewer CPs at higher levels of parental monitoring among individuals with high EXT PGS [IRR = 0.97, 95% CI (0.95, 0.99), *p* = 0.009; Figure 1]. In other words, higher levels of parental monitoring buffered the effect of higher genetic risk.

**Table 1:** Conduct problems in selected EXT PGS-parental monitoring groups and associated comparisons.

EXT PGS	Parental monitoring	*N*	Mean	SD	Range
High	High	221	1.26	0.63	1–6
High	Low	336	2.01	1.37	1–7
Low	High	293	1.20	0.50	0–4
Low	Low	218	1.44	0.83	1–6
Comparisons					
Group 1	Group 2	t	df	*p*	Cohen's *d*
High/high	High/low	−7.68	555	<0.0001	0.70
High/high	Low/high	1.23	512	0.22	0.11
High/high	Low/low	−2.55	437	0.01	0.24
High/low	Low/high	9.67	627	<0.0001	0.79
High/low	Low/low	5.58	552	<0.0001	0.50
Low/high	Low/low	−4.07	509	<0.0001	0.35

High = top 25%; low = bottom 25%; Cohen's *d* = (M_1_ − M_2_)/SD_pooled_; effect size conventions: 0.20 = small, 0.50 = moderate, 0.80 = large.

**Table 2:** Parameter estimates from the main effect and interaction models.

Predictor	IRR	95% CI	Wald *X*^2^	*p*
Main effect model				
Intercept	1.43	1.39–1.46	765.30	<0.0001
EXT PGS	1.07	1.05–1.10	31.68	<0.0001
Parental monitoring	0.86	0.84–0.88	145.90	<0.0001
Interaction model				
Intercept	1.42	1.39–1.46	741.71	<0.0001
EXT PGS	1.07	1.04–1.10	26.22	<0.0001
Parental monitoring	0.87	0.85–0.89	130.90	<0.0001
EXT × parental monitoring	0.97	0.95–0.99	6.85	0.009

EXT PGS, externalizing polygenic score; IRR, incidence rate ratio.

## Discussion

The results presented here indicate that parental monitoring is protective against increased genetic risk for CPs. This finding underscores the potential of family-based prevention and intervention efforts, particularly for children at increased genetic risk (20). One limitation of the current study is that both the EXT GWAS summary statistics and ALSPAC analyses only include individuals from European-like genetic similarity groups. While this limitation reflects a broader issue in the field of statistical genetics, wherein genetic data has not been collected from multi-ancestry groups at the same rate as European-like individuals, future research should aim to replicate these findings in diverse samples and with multi-ancestry GWAS summary statistics. Efforts are currently underway to perform an EXT GWAS in a multi-ancestry population and the results of these analyses will improve our ability to study the genetic component of externalizing in more diverse samples.

The identification of modifiable targets that impact the expression of genetic risk, such as parenting practices, is especially important with the growing provision of genetic feedback. With the rapid proliferation of direct-to-consumer genetic testing, laypeople are receiving information about their genetic risk for biomedical and behavioral problems at higher rates than ever, making it increasingly urgent to study the return of genetic information (21). PGSs for psychiatric and substance use outcomes now perform as well as PGSs already in use in other areas of medicine.^1^ The PGS for externalizing studied here is the most powerful PGS for any behavioral outcome to date, accounting for more variance than many socioenvironmental risk factors. Further, combining the PGS with behavioral and environmental risk indices powerfully differentiates individuals at low and high risk, and studies are underway to test how the delivery of personalized risk profiles influences behavior change (22). Accordingly, identifying actionable targets that mitigate the associations between genetic risk and adverse outcomes can help pave the way for personalized prevention and intervention.

## Data Availability

The data analyzed in this study were obtained from ALSPAC, the following licenses/restrictions apply https://www.bristol.ac.uk/alspac/researchers/access/. Requests to access these datasets should be directed to ALSPAC, alspac-data@bristol.ac.uk.

## Footnotes

1 Dick DM, Dash GF, Hung I, Horgan A. Are polygenic scores for psychiatric and substance use outcomes ready for clinical application? *Curr State Next Steps.* (2025).
